# Supplementary material for: Coronavirus Disease 2019-Associated Thrombotic Microangiopathy: A Single-Center Experience
Source: Int J Mol Sci. 2024 Nov 20;25(22):12475. doi: 10.3390/ijms252212475 (PMC11594656; doi:10.3390/ijms252212475)
Supplement: Supplementary file 1 [file ijms-25-12475-s001.zip › ijms-3287501-supplementary.pdf]

Supplementary Table. Patients characteristics, treatment and outcomes.

Patient	Leading clinical symptoms/signs at first referral	Basic laboratory results at admission to hospital	ADAMTS-13 activity	Functional complement analysis	Immunoserological analysis	Basic laboratory results on discharge from hospital and/or outpatient visit	Outcome
**Pt#1 F, 35y**	Headache, spontaneous mucosal bleeding, suffusions, severe thrombocytopenia.	15.09.2022: Lkci 4,2 10^9/L; Erci 3,08 10^12/L; Hb **82** g/L; Tr **37** 10^9/L; BUN 3,4 mmol/l; Creatinine 79 µmol/l; eGF (CKD-EPI)/1,73 m2 84 ml/min; Bilirubin tot. 28 µmol/l; Bilirubin dir. 10 µmol/l; Albumin 46 g/l; LDH **7,41** µkat/l; Shistocytes **7,8**%.	**1,7** %.	CP activity (haemolitical test): 144 %; AP activity (ELISA AP): 111 %; LP activity: 104 %; Complement component factor B:**425** mg/l; factor H: 514 mg/l; factor I: 32 mg/l; C3: 1,09 g/l; C4: 0,28 g/l; SC5b-9 litic complex: 141 ng/ml; Urinary SC5b-9 litic complex: **50** ng/ml.	ANA negative; anti-ENA-Sm,U1RNP,Ro,La,Scl-70,Jo-1,PCNA,PM/Scl,SL,Ku negative; aCL IgG <5, IgM <5; anti-beta2GPI IgG <2, IgM <2, IgA <2; aPS/PT IgG <5, IgM <5, IgA <5.	20.09.2022: Lkci 9,5 10^9/L; Erci 3,08 10^12/L; K-Hb **87** g/L; Tr 249 10^9/L; BUN 5,5 mmol/l; Creatinine 77 µmol/l; eGF (CKD-EPI)/1,73 m2 86 ml/min; Bilirubin tot. 7 µmol/l; Bilirubin dir. 3 µmol/l; Albumin 41 g/l; LDH 2,38 µkat/l.	Complete resolution of symptoms/signs of the disease.
**Pt#2 M, 43y**	Malignant hypertension (RR>230/125 mmHg), retinal haemorrhages, acute kidney injury.	23.02.2021: Lkci 10,0 10^9/L; Erci 3,48 10^12/L; K-Hb **108** g/l; Tr **106** 10^9/L; BUN **26,4** mmol/l; Creatinine **536** µmol/l; eGF (CKD-EPI)/1,73 m2 **10** ml/min; Bilirubin tot. **23** µmol/l; Bilirubin dir. 6 µmol/l, Albumin g/l 41; LDH **5,18** µkat/l.	45,1 %.	CP activity (haemolitical test) 103 %; AP activity (ELISA AP): 104 %; LP activity 170 % ; Complement component factor B: **402** mg/l; factor H: 376 mg/l; factor I: 35 mg/L; C3: 0,90 g/l; C4: 0,29 g/l; SC5b-9 litic complex: 236 ng/ml; C3Nef: 5,1% - negative; Antibodies against factor H (anti-FH): 2,6 U/ml – negative, Antibodies against C1q (anti-C1q):2,7 U/ml - negative.	anti-ds DNA negative; **ANA positive**, titer **1:640**; anti-ENA-Sm,U1RNP,Ro,La,Scl-70,Jo 1,PCNA,PM/Scl,SL,Ku negative; anti-CCP <25; aCL IgG **6**, IgM <5, IgA <5; anti-beta2GPI IgG <2, IgM <2, IgA <5.	6.4.2021: Lkci 10,8 10^9/L; Erci 4,51 10^12/L; K-Hb 139 g/L; Tr 249 10^9/L; BUN **23,1** mmol/l; Creatinine **727** µmol/l*; eGF (CKD-EPI)/1,73 m2 **7** ml/min*; Bilirubin tot. 6 µmol/l; Bilirubin dir. 2 µmol/l; Albumin 46 g/l; LDH 3,44 µkat/l. *dialysis dependant	Resolution of symptoms/signs of malignant hypertension, ESRD. Kidney transplant (deceased donor) July 2022, no TMA recurrence.

**Pt#3 M, 32y**	Nausea, vomiting, oliguric acute kidney injury.	29.09.2021 Lkci 4,6 10^9/L; Erci 2,79 10^12/L; K-Hb **86** g/L; Tr **120** 10^9/L; BUN **19,2** mmol/l, Creatinine **214** µmol/l; eGF (CKD-EPI)/1,73 m2 **34** ml/min; Bilirubin tot. **27** µmol/l; Bilirubin dir. 9 µmol/l; Albumin 35 g/l; LDH **7,74** µkat/l.	74,8 %.	CP activity (haemolitic test): 102 %; (AP activity (ELISA AP): 82 %; LP activity: 13 %; complement component factor B: 277 mg/l; factor H: 378 mg/l; factor I: 24 mg/l; C3: 0,7 g/l; C4: 0,14 g/l; SC5b-9 litic complex: 198 ng/ml; Urinary C5b-9 litic complex: 8 ng/ml.	ANA negative; anti-ENA - Sm,U1RNP,Ro,La,Scl-70,Jo-1,PCNA,PM/Scl,SL,Ku negative; aCL IgG <5, IgM <5; anti-beta2GPI IgG <2, IgM <2, IgA <2; aCl negative; ANCA negative.	4.10.2021 Lkci 3,97 10^9 /L; Hb **73** g/L;Tr 282 10^9 /L; BUN 7,1 mmol/l; Creatinine **126** µmol/l; eGF (CKD-EPI)/1,73 m2 **65** ml/min; LDH **5,02** µkat/l.<br><br>**29.10.2021:** Lkci 4,7 10^9/L; Erci 3,90 10^12/L; Hb 122 g/L; Tr 173 10^9/L; BUN 3,7 mmol/L; Creatinine 96 µmol/L; eGF (CKD-EPI)/1,73 m2 90 mL/min; Bilirubin tot. 15 µmol/L; Bilirubin dir. 4 µmol/L; Albumin 46 g/L.	Complete resolution of symptoms/signs of the disease.
**Pt#4 M, 33y**	Malignant hypertension (RR 258/148 mmHg), blindness (retinal haemorrhages and edema of the optic nerve papilla), acute kidney injury, nausea, vomiting, severe thrombocytopenia, hemolytic anaemia.	14.10.2021 Lkci 6,7 10^9/L; Erci 2,10 10^12/L; Hb **66** g/l; Tr **29** 10^9/L; BUN **46,2** mmol/l; Creatinine **819** µmol/l; eGF (CKD-EPI)/1,73 m2 **7** ml/min; Bilirubin tot. 23 µmol/l; Bilirubin dir. 7 µmol/l; Albumin 36 g/l; LDH **26,70** µkat/l; Shistocytes **6,7**%.	57,1 %.	CP activity (haemolitic test): 127 %; AP activity (ELISA AP): 115 %; LP activity 25 %; Complement component factor B: 314 mg/l; factor H: 439 mg/l; factor I: 29 mg/l; C3: 0,71 g/l; C4: 0,22 g/l; SC5b-9 litic complex 182 ng/ml; Urinary C5b-9 litic complex >**181** ng/ml; C3Nef: 0 % - negative; Antibodies against factor H (anti-FH): 0,7 U/ml – negative; Antibodies against C1q (anti-C1q): 1,3 U/ml – negative.	ANA negative; anti-ENA-Sm,U1RNP,Ro,La,Scl-70,Jo-1,PCNA,PM/Scl,SL,Ku negative; aCL IgG <5, IgM <5; anti-beta2GPI IgG <2, IgM <2, IgA <2; aPS/PT IgG <5, IgM <5, IgA <5; ANCA negative.	15.11.2021 Lkci 5,6 10^9/L; Erci 3,70 10^12/L; Hb 119 g/l; Tr **12** 10^9/L; BUN 28,1; Creatinine **806** µmol/l*; eGF (CKD-EPI)/1,73 m2 **7** ml/min*; Bilirubin tot. 8 µmol/l; Bilirubin dir 2 µmol/l; Albumin 47 g/l; LDH 3,98 µkat/l. *dialysis dependant	Improvement of blood pressure and hematologic disease, severe visual impairment persists, ESRD.
**Pt#5 M, 25y**	Nausea, vomiting, diarrhoea, acute kidney injury,	10.1.2022	49,2%.	CP activity (haemolitic test): 114 %; AP activity	ANA negative; anti-ENA-Sm,U1RNP,Ro,La,Scl-70,Jo-	24.02.2022 Lkci 11,28 10^9/L; Hb **108** g/l; Erci 3,51 2,10 10^12/L;	Improvement of gastrointestinal symptoms and hematologic disease,

	thrombocytopenia,<br>hemolytic anaemia.	Lkci 4,0 10^9/L; Erci 2,33 2,10 10^12/L; Hb **72** g/l; Tr **58** 10^9/L; BUN **47,3** mmol/l; Creatinine **1313** µmol/l; eGF (CKD-EPI)/1,73 m2 **4** ml/min; Bilirubin tot. 5 µmol/l ; Bilirubin dir. 2 µmol/l; Albumin 33 g/l; LDH **7,14** µkat/l; Shistocytes **4**%.		(ELISA AP): 112 %;<br>LP activity: 125 %;<br>complement component factor B: **457** mg/l;<br>factor H: 436 mg/l;<br>factor I: 42 mg/l;<br>C3: 0,93 g/l;<br>C4: 0,23 g/l;<br>C5b-9 litic complex**: 350** ng/ml;<br>C3Nef: 3,6 %-negative;<br>Antibodies against factor H (anti-FH): 2,8 U/ml-negative;<br>Antibodies against C1q (anti-C1q): 5,2 U/ml-negative.	1,PCNA,PM/Scl,SL,Ku negative;<br>aCL IgG <5, IgM <5;<br>anti-beta2GPI IgG <2, IgM <2, IgA <2;<br>aPS/PT IgG <5, IgM <5, IgA <5;<br>ANCA negative.	Tr **121** 10^9/L;<br>BUN **27,2** mmol/l; Creatinine **679** µmol/l *****;<br>eGF (CKD-EPI)/1,73 m2 **9** ml/min*****; Bilirubin tot. 7 µmol/l; Bilirubin dir. 3 µmol/l; Albumin 35 g/l; LDH **5,48** µkat/.<br>*dialysis dependant	ESRD.<br><br>Kidney transplant (deceased donor) April 2024- no TMA recurrence.
**Pt#6**<br>**M, 52y**	Nausea, propulsive vomiting, severe worsening of chronic kidney transplant disease* (uremia)<br>*pt with transplanted kidney, creatinine at last follow up before referral was 181 µmol/l, eGF 36 ml/min.	26.2.2021<br>Lkci 5,4 10^9/L; Erci 2,82 10^12/L; Hb **80** g/L; Tr 179 10^9/L; BUN **15,2** mmol/L; Creatinine **688** µmol/l******; Bilirubin tot. 10 µmol/L; Bilirubin dir. 6 µmol/L; Albumin 30 g/L; LDH 3,24 µkat/l; Tacrolimus 4,5 µg/l.<br>** hemodialysis performed in the emergency room before admission due to uremia (Creatinine>2000 µmol/l)	Not performed.	Not performed.	anti-CCP <25;<br>aCL IgG <5, IgM <5; IgA <5;<br>anti-beta2GPI IgG <2, IgM <2, IgA <2;<br>aPS/PT IgG <5, IgM <5, IgA <5.	26.4.2021<br>Lkci 6,1 10^9/L; Erci 3,11 2,82 10^12/L; K-Hb **98** g/l; Tr 154 10^9/L; Tacrolimus 6,5 µg/l; BUN **25,2** mmol/l; Creatinine **520** µmol/l;<br>eGF (CKD-EPI)/1,73 m2 **10** ml/min; Bilirubin tot. 13 µmol/l; Bilirubin dir. 5 µmol/l; Albumin 34 g/l; LDH 4,09 µkat/l.	Partial recovery of kidney graft function - at the time of discharge he was not on dialysis.<br>Two months later ESRD, start of dialysis.<br>2nd Kidney transplant (deceased donor) August 2024, no TMA recurrence.
**Pt#7**<br>**M, 33y**	Fever, cough, diarrhoea,<br>signs of malignant hypertension (RR 210/110), visual	30.8.2024<br>Lkci 11,7 10^9/L; Erci 2,81 10^12/L; Hb **87** g/L; Tr **78** 10^9/L;<br>BUN **32,2** mmol/L; Creatinine **1072**	80,1 %.	CP activity (haemolitic test): 94 %;<br>AP activity (ELISA AP): 96 %;<br>LP activity: 27 %;<br>Complement component factor B: 238 mg/l;	RF <15,9 IU/mL-negative;<br>ANCA negative;<br>Anti-GBM negative;<br>Anti-C1q negative;<br>Anti-PLA2R IgG negative;<br>Anti-THSD7A IgG negative;	13.9.2024<br>Lkci 12,7 10^9/L; Erci 2,82 10^12/L; Hb **88** g/l; Tr 177 10^9/L;<br>BUN **27,8** mmol/l; Creatinine **803** µmol/l *****;	Resolution of symptoms/signs of malignant hypertension,<br>ESRD.

	disturbances, acute kidney injury.	µmol/L; eGF (CKD-EPI)/1,73 m2 **5** mL/min; Bilirubin tot. 10 µmol/L; Bilirubin dir. 4 µmol/L; LDH **8,15** µkat/L; Haptoglobin **<0,07** g/l.		factor H**: 677** mg/l; factor I: 35 mg/l; C3: 0,74 g/l; C4: 0,27 g/l; SC5b-9 litic complex: 121 ng/ml; Bb: **3,03** µg/ml; antibodies against factor H (anti-FH): 1,75 U/ml -negative; C3Nef: 4,02 % -negative; Antibodies against C1q (anti-C1q): 1,18 U/ml-negative.	ANA negative; anti ENA-Sm,U1RNP,Ro,La,Scl-70,Jo-1,PCNA,PM/Scl,SL,Ku negative; aCL IgG <5, IgM <5; IgA <5; anti-beta2GPI IgG <2, IgM <2, IgA <2; aPS/PT IgG <5, IgM <5, IgA <5; lupus anticoagulants-negative.	eGF (CKD-EPI)/1,73 m2 **7** ml/min*****; Bilirubin tot. 5 µmol/l; Bilirubin dir. 2 µmol/l; Albumin 38 g/l; LDH 3,32 µkat/l. *dialysis dependant	
**Pt#8 F, 29y**	Fatigue, high blood pressure (180/110 mmHg), acute worsening of chronic kidney disease - at the last follow-up examination she had a Creatinine of 117 µmol/l, eGF 55 ml/min.	13.8.2024 Lkci 4,2 10^9/L; Erci 2,75 10^12/L; K-Hb **83** g/l; Tr 152 10^9/L; BUN **27,7** mmol/l; Creatinine **720** µmol/l; eGF (CKD-EPI)/1,73 m2 **6** ml/min; Bilirubin tot. 12 µmol/l; Bilirubin dir. 3 µmol/l; Albumin 37 g/l; LDH 3,92 µkat/; Haptoglobin 1,30 g/l, Shistocytes **2,1**%.	Not performed	CP activity (haemolitic test): 139 %; AP activity (ELISA AP): 93 %; LP activity: 79 %; complement component factor H: 562 mg/l; factor I: 29 mg/l; factor B: 240 mg/l; C3: 0,94 g/l; C4: 0,22 g/l; SC5b-9 litic complex: 186 ng/ml; Bb: **1,95** µg/ml.	ANA negative; anti-ENA-Sm,U1RNP,Ro,La,Scl-70,Jo-1,PCNA,PM/Scl,SL,Ku negative; antibodies associated with SSc (Scl-70 <5 AU; CENP-A <5 AU; CENP-B <5 AU; PM-Scl 100 <5 AU; PM-Scl 75 <5 AU; Ku <5 AU; RNA Polimeraza III <5 AU; RNP 68kD/A/C <5 AU; Th/To <5 AU; Fibrilarin <5 AU; NOR-90 <5 AU; SSA/Ro52kD <5 AU)-negative.	23.8.2024 Lkci 7,5 10^9/L;-Erci 2,88 10^12/L; Hb **87** g/L; Tr 197 10^9/L; Haptoglobin 1,62 g/L; BUN **31,6** mmol/L; Creatinine **771** µmol/L; eGF (CKD-EPI)/1,73 m2 **6** mL/min; LDH 3,58 µkat/L.	Improvement of high blood pressure, ESRD. On hemodialysis since 6.9.2024.

Legend of abbreviations and reference values of selected laboratory parameters (significant deviations in laboratory values are highlighted):
ANA-antinuclear antibodies, ENA-extractable antibody nuclear panel, aCl-anticardiolipin antibodies, anti-beta2GPI- Beta 2 glycoprotein I antibodies, aPS/PT-antithrombin antibodies, ANCA- anti neutrophil cytoplasm antibodies,

CP-classical complement pathway (normal value for adults: 50-150%),
AP-alternative complement pathway (normal value for adults: 30 - 113%),
LP-lectin complement pathway (normal value for adults: 0 - 125%),
C3-complement component C3 (reference value for adults 0,60 - 1,30 g/l),
C4-complement component C4 (reference value for adults 0,10 - 0,30 g/l),
C5b-9-membrane attack complex, litic complex (reference value for adults in plasma: 127 - 303 ng/ml; reference value for adults in urin: < 30 ng/ml),

Factor B-(reference value for adults 205-400 mg/l),
Factor H-(reference value for adults 380,6-674,9 mg/l),
Factor I-(reference value for adults 21,2-42,1 mg/l),
Bb- (reference value for adults 0,49 - 1,42 μg/ml),

LDH-lactate dehidrogenase (normal value <4,13 μkat/l),

Bilirubin tot.-total bilirubin (normal value<15 μmol/l),

Bilirubin dir.-direct bilirubin (normal value<5 μmol/l,

Haptoglobin (normal value 0,3-2,0 g/
